# Should the Genitoplasty of Girls with CAH be Done in One or Two Stages?

**DOI:** 10.3389/fped.2013.00054

**Published:** 2014-01-10

**Authors:** Ricardo González, Barbara M. Ludwikowski

**Affiliations:** ^1^Pediatric Surgery and Urology, Auf der Bult Kinder – und Jugendkrankenhaus, Hannover, Germany

**Keywords:** disorders of sex development, congenital adrenal hyperplasia, vaginoplasty, feminizing genitoplasty, DSD, CAH

The debate over the timing of surgical repair in girls with congenital adrenal hyperplasia (CAH) is intense and was in part started by patient advocacy groups largely composed of dissatisfied adults operated in childhood. Their influence has been beneficial to make surgeons reconsider gender reassignment in certain cases of DSD and other genital malformations such as penile agenesis and cloacal exstrophy. Some authors have proposed a ban on all no-medically necessary surgery in infants (1).

However, we think this debate as useful as it may be should not include the treatment of girls with CAH. One must keep in mind that girls with 21-hydroxylase deficiency, the most common form of CAH, are genotypically female, and have a normal potential for fertility and sexual function, and to date there have been no instances of gender dysphoria reported in this population. The situation is quite similar to that of proximal hypospadias in boys, a condition that, as is universally accepted, is best repaired in infancy. If we have to ban early genital reconstruction in girls with CAH, surgeons must be rational and consistent in their behavior and also ban hypospadias repair (not to speak of most circumcisions) in infants and children who cannot give consent.

Two recent articles in Frontiers in Pediatrics (2, 3) advocate or suggest seriously considering the surgical correction of the genital ambiguity in girls with CAH, in two stages: one early stage, to feminize the appearance of the external genitals, and vaginoplasty as a second stage after puberty. The rationale for this approach is the high reported incidence of post-pubertal vaginal (3) introital stenosis when the vaginoplasty was done in childhood (4–8). The validity of this proposal has not been tested. In fact there is only one report suggesting that vaginoplasty done after puberty carries a lower rate of stenosis (9). On the other hand, several reports indicate that the stenosis detected after puberty is relatively easy to repair (5, 8).

But the two-stage proposal to repair CAH in no way addresses the debate on infant genital surgery since it advocates early vulvo and clitoroplasty and only assumes that post-pubertal vaginoplasties will have a better outcome than when done in infancy.

The report by Hoepffner et al. (9) on which the recommendation to stage the repair is based is worth close analysis. The authors reviewed 46 patients with CAH aged between 16 and 46 years. Of interest are the 35 women who had a Prader stage of virilization 3 or greater. Most patients had a Fortunoff and Lattimer flap vaginoplasty, two had a vaginal pull through (10), and three had no vaginoplasty. No patient had undergone en-block urogenital mobilization with a posterior flap (11). Thirteen patients had the vaginoplasty at or before the age of 12 years (mean age 8.6, range 2–12) and 19 had it at a mean age of 14.3 years (range 13–27). We used a cut off age of 12 for this analysis since no Tanner stage at the time of vaginoplasty is given in the article. Patients were evaluated by a questionnaire and when not sexually active, a gynecological examination was performed to determine the possibility of having sexual intercourse.

Of the 13 patients with a prepubertal vaginoplasty, 6 required a revision after puberty whereas only 1/19 in whom the vaginoplasty was done later needed a revision. Nevertheless, in the end there were no differences between the groups in the ability to have intercourse or having the potential for doing so suggesting that the revisions were successful. In fact, the only four patients who were thought to have a vagina inadequate for intercourse had no vaginoplasty (two) or had it at the age of 15 years (two). Both of these women were classified as Prader 5.

Our policy and recommendations are to perform the repair of the genital in CAH when the infant is endocrinologically stable. This is consistent with our policy to repair hypospadias in infancy. In our experience, two-stage surgery has some disadvantages. After the initial vulvo and clitoroplasty, it remains scarring in the area of the future creation of the vaginal introitus. The distal urogenital sinus, which is so useful to create a mucosa-lined vestibule, is often disturbed to the point of making it unavailable at the time of vaginoplasty. For this reason, we favor early one-stage reconstruction. The options available as well of the existing controversies should be thoroughly explained to the parents. If the parents opt for early surgery, they should be informed that an examination under anesthesia is recommended at 3 months and necessary at puberty.

The operation consists of a vulvoplasty and vaginoplasty by a Fortunoff flap or en-block mobilization according to the Prader stage. If the degree of clitoral hypertrophy so demands, we do a reduction of the corpora cavernosa with preservation of the dorsal neurovascular bundle (12). The glans clitoris is not reduced but simply hidden by creation of a clitoral hood. We perform a brief examination under anesthesia 3 months later to assess the early result and then perform an examination under at puberty to assess the vaginal introitus in a non-traumatic way before the onset of sexual activity and recommend revision or dilatation when needed. This approach seems to us to be as valid as the proposed two-stage procedures.

## Conflict of Interest Statement

The authors declare that the research was conducted in the absence of any commercial or financial relationships that could be construed as a potential conflict of interest.
